# Books Reviewed

**Published:** 1877-03

**Authors:** 


### Books Reviewed.

*A Treatise on Surgery, its Principles and Practice* By T. HOLMES, M. A., Contab., etc. Philadelphia: Henry C. Lea, 1876.

Mr. Holmes says in his Preface that this volume is the result of an attempt to represent the present condition of Surgery as taught in England, by a treatise which shall not be unworthy to rank with the other excellent text-books in use in the schools. The work was also undertaken that it might be to some extent an introduction to the more elaborate System of Surgery of which he is the Editor. The well known ability of the author as a surgeon and as a writer on surgical topics will be as excellent an introduction as he could desire for his work.

The opening chapter is upon inflammation and the process of union in soft parts, traumatic fever and dressing of wounds. Under the latter head the author announces his preference for the method of Lister, at the same time expressing the opinion that the opponents of the germ theory have the best of the argument. Mr. Holmes cites an instance which illustrates the value of this method, and which at the same time bears upon a point which we have seen alluded to by a recent writer, whose name at this moment escapes our memory. We refer to the value of the carbolic spray which Mr. Lister deems so essential to the proper dressing of wounds. The case cited by Mr. Holmes is one of compound fracture of both bones of the leg, in the treatment of which it was found necessary to resect more than two inches of both bones. Under Lister's method no traumatic fever was experienced and the wouud healed rapidly and kiudly. It strikes us that prior to the operation abundant opportunity had been given for the access of whatever agent is provocative of inflammation, to the wound as much as there would be during any of the subsequent dressings and that it would be as easy to cleanse the wound at the time of dressing and prevent the entrance of putrefactive agents, disease germs or otherwise, as at the original operation, without resorting to the trouble of the spray or "antiseptic veil." Certainly no spray was playing on the wound from the moment of its reception up to the time of the operation, and yet no serious results followed. Why, therefore, if the same precautions are taken to cleanse the wound prior to the application of dressings, is it essential to keep a constant spray playing during the opening of an abscess or the dressing of a wound?

We have neither the time nor space to notice this work as it deserves. We can simply say that from a somewhat careful examination, we have formed a high estimate of its merits as a text-book. B.

---

*Cyclopædia of the Practice of Medicine.* Edited by Dr. H. Von Ziemssen. Vol. XI. *Diseases of the Peripheral Cerebro Spinal Nerves.* By Prof. Wilhelm Heinrich, Erb., Translated by Mr. Henry Power, of London. Albert H. Buck, M. D., Editor American Edition. New York: William Wood & Co., 1876. Buffalo: H. Matteson, Agent. (Subscription only.)

This work treats of several diseases which are but little understood, although of frequent occurrence. The author holds to the division of diseases of the peripheral nerves into functional and anatomical. Under the first are included neuralgia, hyperæsthesia, anæsthesia, paralysis, spasm, etc. Under the latter division he includes hyperæenia, atrophy, inflammation, etc. Over one hundred and seventy pages are devoted to a very interesting discussion of neuralgia. Under Etiology the author makes some interesting remarks upon predisposition to certain neuroses and touches upon the fact that heredity has a large place in the etiology of many nervous diseases. We are pleased with the author's remarks upon this topic and only wish that they could be read by every physician. In the treatment of neuralgia the author speaks very highly of electricity. The faradic current he uses chiefly in peripheric neuralgia. In these, the writer has seen some most admirable effects from the use of the rapidly interrupted faradic current. He has also found it to be of value, combined with the galvanic current, in the treatment of neuralgic pains the result of lead poisoning. Some concise rules are given by the author as to the modes of applying electricity.

The author also dwells to some extent upon the uses of electricity in the differential diagnosis of various nerve lesions, and in the examination of paralyzed nerves and muscles.

The article on paralysis is of special interest as is also that upon "cramps," that common and oft painful affection which either from carelessness or ignorance of its importance in many instances is but little understood.

The volume as a whole is one of the most interesting of the series, and its careful perusal by physicians is recommended. B.

*A Century of American Medicine*—1776–1876. By Edward H. Clarke, M. D., late Professor of Materia-Medica in Harvard University, etc.; Henry J. Bigelow, M. D., Professor of Surgery in Harvard Uniuersity, etc.; Samuel J. Gross, M. D.; D. C. L. Oxŏn, Professor of Surgery in the Jefferson Medical College, Phi[adelphia, etc.; T. Gailord Thomas, M. D., Professor of Obstetrics, etc., in the College of Physicians and Scrgeons, New York, etc., and J. S. Billings, M. D., Librarian of the National Medical Library, Washington, D. C. pp. 366. Philadelphia: Henry C. Lea, 1876.

This book is a reprint from papers which have recently appeared in the "American Journal of the American Sciences," and at once adapted to the wants of the careful student, the busy practitioner, and the intelligent non-professional reader. The distinguished names upon its title page are a guaranty of its intrinsic excellence. Its small size and moderate price bring it within the reach of all. It is full of rich suggestions and is an epitome of wisdom, aside from its great historical interest and value, and is a timely, appropriate, and much needed contribution to the literature of to-day. We see but one omission, or addition which might still further insure its usefulness, and that is a lack of marginal dates, for which there is ample room, and which would be a great convenience to the careful student.

Dr. Clarke's paper is upon "Practical Medicine;" Dr. Bigelow's, "A History of the Discovery of Anæsthesia;" Dr. Gross's, upon "Surgery;" Dr. Thomas treats of "Obstetrics and Gynæcology" and Dr. Billings of "Literature and Institutions."

Taken together these papers form a book which is surely interesting, comprehensive and instructive, and should be very generally read, being of exceeding value both to those within and without the profession.

---

*A Treatise on Hernia: With a New Process for its Radical Cure, and Original Contributions to Operative Surgery, and New Surgical Instruments.* By Greensville Dowell, M. D., Professor of Surgery in Texas Medical College, etc., etc. Philadelphia, D. G. Brinton, 1876.

The author of this work evidently believes in demonstrating the truth of the maxim, "of making many books, there is no end," and without a very great show of "rhyme or reason," puts forth a "Treatise" on Hernia. About thirty pages are devoted to the description of Hernia, but they present no facts which cannot be better treated in almost any work on Surgery. A student attempting to gain information from this portion of the work would be led into inevitable confusion by the many terms by which the author defines the various varieties of hernia, as, for instance, infundibulatic, diverticulatic, intusceptic, ligmentatic, etc.

The author's method, briefly stated, consists in surrounding the canal by one or more wire ligatures, as the case demands, which are tied over a roll of adhesive plaster or other substance, so as to approximate the sides of the canal. The ligatures are allowed to remain eight days. The wire is made to pass into the peritoneal cavity, which, it would seem, adds materially to the dangers of the operation. To facilitate the passage of the wire the author employs a double pointed curved needle with an eye in each extremity.

Dr. Dowell, on page sixty-seven, informs us that he has, up to Sept. 12th, operated on sixty-eight cases with sixty cures; to these may be added one case operated on Sept. 13th, reported as successful. This is certainly a very remarkable result, and suggests a little examination of the cases. Cases 13, 14 and 15, are reported on the pages open before us (66, 67). Case 13 was operated on April 19, 1876, Case 14, the same date, Case 15, on May 26; on August 28th, a little over *four months* from the time of the first operation, they are called perfect cures; certainly the time which had elapsed since the operation, was too brief to determine the final result in these cases. We think that if Dr. Dowell will examine his cases at the end of a little longer time, he will find that the result is not as good as he anticipated, and that his statistics will have to be materially altered. The original contributions do not constitute anything of note.

---

*Studies, Chiefly Clinical, on the Non-Emetic use of Ipecacuanha. With a Contribution to the Therapeutics of Cholera.* By Alfred Woodhull, M. D., Asst. Surgeon U. S. Army. Philadelphia, J. B. Lippincott & Co., 1876. Buffalo, Peter Paul & Bro.

The object of this essay is to call attention to the value of Ipecacuanha as a therapeutic agent, aside from its emetic properties. Dr. Woodhull advances the statement that it is a nervous stimulant acting chiefly upon the sympathetic system. He has evidently given the subject considerable attention, and has carefully searched authorities for evidence of its uses as a non-emetic. A long list of affections are cited in which it has proven more or less useful, beginning with dysentery. The writer has had somewhat gratifying evidences of its value in this disease, in Hospital practice, but has never administered it in as large doses as are used by Dr. Woodhull.

The value of Ipecacuanha in bowel and gastric troubles we think, in all probability, is not fully appreciated, and there are other affections, doubtless, in which it will prove of use when administered in sufficiently large doses, but whether the author's high opinion of it will ever be fully endorsed, time and careful trial alone can tell.

